# Keeping an Eye on Decellularized Corneas: A Review of Methods, Characterization and Applications

**DOI:** 10.3390/jfb4030114

**Published:** 2013-07-10

**Authors:** Samantha L. Wilson, Laura E. Sidney, Siobhán E. Dunphy, James B. Rose, Andrew Hopkinson

**Affiliations:** Division of Ophthalmology and Visual Sciences, University of Nottingham, Queens Medical Centre, Nottingham, NG7 2UH, UK; E-Mails: samantha.wilson@nottingham.ac.uk (S.L.W.); laura.sidney@nottingham.ac.uk (L.E.S.); paxsd2@nottingham.ac.uk (S.E.D.); paxjr@nottingham.ac.uk (J.B.R.)

**Keywords:** decellularization, extracellular matrix, cornea, tissue engineering

## Abstract

The worldwide limited availability of suitable corneal donor tissue has led to the development of alternatives, including keratoprostheses (Kpros) and tissue engineered (TE) constructs. Despite advances in bioscaffold design, there is yet to be a corneal equivalent that effectively mimics both the native tissue ultrastructure and biomechanical properties. Human decellularized corneas (DCs) could offer a safe, sustainable source of corneal tissue, increasing the donor pool and potentially reducing the risk of immune rejection after corneal graft surgery. Appropriate, human-specific, decellularization techniques and high-resolution, non-destructive analysis systems are required to ensure reproducible outputs can be achieved. If robust treatment and characterization processes can be developed, DCs could offer a supplement to the donor corneal pool, alongside superior cell culture systems for pharmacology, toxicology and drug discovery studies.

## 1. Introduction

Corneal blindness encompasses a complex profile of clinical indications, all presenting with a loss of functional vision that affects millions of people worldwide [1,2,3,4]. Blindness such as this can have numerous causes and can range in severity, but in most cases corneal graft surgery is the most viable treatment option [5]. The use of cadaveric donor corneal grafts (allografts) for transplantation is routine in current clinical practice. The introduction of tissue quality controls and donor screening has improved success rates of transplantation operations, making corneal transplantation the most successful human tissue transplantation procedure [3,6,7]. Despite this, 1 in 6 full thickness corneal transplants experience some degree of rejection [8]. 

Arguably, the biggest current limitation to corneal transplantation is the supply of high quality donor tissue [9]. This shortfall differs drastically between territories, with westernized nations generally well provided for [10,11], and demand in Africa and Asia considerably outstripping supply [11,12]. The increasing worldwide trend for refractive surgery procedures [10,11], cultural and religious concerns related to the use of cadaver corneas [11], and the short shelf-life of suitable corneas [13], all add to tissue shortages, resulting in over 10 million untreated patients globally [14]. 

The current and projected donor shortages are a driver for many to develop feasible long-term alternatives to cadaveric corneal donor tissue. Ideally, an alternative should be equivalent, preferably superior to cadaveric donor tissue. In order to achieve this, many demands must be met. A corneal tissue equivalent needs to be biocompatible; have the spatial architecture of the native tissue so that it is optically transparent; be strong to withstand manipulation in culture, potential suturing, irrigation and handling during surgery; have a flexible structure so that it can take the shape of the eye and lay flat on the surface and also have the ability for oxygen and nutrient transfer through the structure. The manufacturing process needs to be easily reproduced with consistent quality, preferably at high speed and low cost.

Alternatives currently in clinical or pre-clinical development can be categorized into the following areas: keratoprostheses (KPros), xenografts, tissue engineered (TE) constructs and more recently decellularized corneas (DCs). A KPro is an acellular synthetic implant that is intended to be biologically, mechanically and functionally attached to the eye [15], permit visible light transmission, whilst protecting the retina from ultraviolet (UV) damage [16]. Current examples of KPros that have undergone clinical trials include AlphaCor™ [17,18,19] and the Boston Type 1 [20]. Xenografts occur when tissue(s) from one species are transplanted into a different species. Many attempts have been made to transplant animal corneas into humans, including the use of cows, dogs, fish, gibbons, pigs, sheep and rabbits [11]. Porcine corneas are most commonly used as they have a similar physiology and refractive properties compared to human corneas and are relatively easy to obtain [21]. However, xenografts for corneal replacement do not have to be of corneal origin. For example, van Essen *et al.* (2013) [22] recently reported the use of fish scales harvested from the Tilapia fish as an alternative tissue source, as the organized collagen fiber arrangement is reminiscent of the human corneal stroma. Tissue engineering (TE) can be described as “*the production of biological or semi-synthetic living tissue for use as replacement tissues for damaged or diseased tissues*” [23]. Often, it is the generation of a synthetic tissue by seeding isolated, specific cells into or onto a template (often referred to as a scaffold) and culturing it in a dynamic environment with the aim to eventually form a tissue which mimics the morphological, physiological and biochemical properties of the natural tissue as closely as possible. However, the resulting construct does not necessarily have to be cellularized. TE corneal replacements can be manufactured using natural materials such as collagen, fibrin, gelatin or alginate, or from synthetic polymers such as poly(ethylene glycol) (PEG), poly vinyl alcohol (PVA) or poly (acrylic acid) (PAA) or a combination of both natural and synthetic materials [24]. 

The advantages and disadvantages of Kpros, xenografts and TE corneas have been summarized in Table 1.

**Table 1:** Alternative techniques to corneal allografting, advantages and disadvantages.

Corneal replacement	Advantages	Disadvantages
**Keratoprostheses**: (KPros) an acellular artificial implant.	Currently the only synthetic corneal replacements with market approval [14]. An alternative treatment for patients considered untreatable by conventional corneal allografting [25,26]. KPro implantation procedure is no more invasive or complex than routine corneal transplantation [27]. KPro procedure is reversible [27]. Clinical data is being accumulated. Scheduling independent of human donor availability.	Success is dependent upon patient maintenance of the device [16]. Many KPro materials are non-cell adhesive and require modification to allow for cell adhesion and migration [28]. Several complications have been seen including: wound leaks [3,20]; inflammation and infection due to protein adhesion [29,30]; increased glaucoma [27,31,32]; extrusion or protrusion of the implant [3,25,29]; tissue melting [3,32]. Some common eye drugs are also harmful to certain types of KPros [19]. Limited long-term success [3]. Limited clinical use [27].
**Xenograf**t: A cellular or acellular tissue graft derived from another species [33].	A virtually unlimited organ, tissue and cell source. Scheduling independent of human donor availability [34]. Porcine corneas are most commonly used and have a similar physiology and refractive properties compared to human corneas and are relatively easy to obtain in large numbers [21] thus, are commercially advantageous [9,11]. Clinical trials using porcine xenografts currently underway.	Commonly used porcine corneas may be unacceptable based on religious beliefs (Islam, Judaism, Jainism) [11]. All xenografts eventually fail due to immune response. Xenografts are rejected more quickly than allograft tissues when similar tissues and circumstances are compared [35]. Risk of cross-species disease transmission. Poor public perception.
**Tissue Engineered (TE) constructs**: a manufactured biological or semi-synthetic constructs that can be cellular or acellular.	Compelling advances in the development of synthetic corneal replacements and culture of human corneal cells onto and within supporting substrates. It has already been shown that the three main corneal layers can be recreated *in vitro* using collagen-based scaffolds and immortalized cell lines [36]. Success in Phase 1 clinical trials have been reported for acellular corneal matrices [14].	Gross measurable results of TE corneas are poor [9,16,37]. Lack of tensile strength to permit surgical manipulation and attachment of the corneal equivalent. Failure to mimic native surface curvature [38]. Lack of the native stromal architecture [39]. Biomechanical and optical properties of the cornea models are often compromised [40]. Presently, there is no cellularized TE corneal equivalent in routine clinical use. No standardized cell sources available.

## 2. Human Decellularized Corneas—A more Promising Alternative?

The drawbacks of allografts, KPros, xenografts and TE corneal constructs have led to the exploration of human DCs as an alternative. A DC is one in which all the cells and cellular components have been completely removed, through chemical, biological or physical methods, leaving a biological scaffold of native extracellular matrix (ECM) proteins. The resulting ECM-derived scaffolds are becoming progressively popular [41,42] as a possible scaffolds for *in vitro* corneal modeling [43,44] and as an alternative tissue source for corneal transplantation. DC matrices are well suited, but not limited to, deep anterior lamellar keratoplasty (DALK) procedures [45]. They have also been investigated as a carrier for *in vitro* expanded human endothelial cells for use in Descemet’s stripping endothelial keratoplasty (DSEK) [46,47]. 

DCs differ from TE corneas or KPros because the native structure is already present, as nature intended; *i.e*., the DC has not been manufactured, synthesized or produced in any manner, as in the case of TE constructs or KPros. An acellular corneal xenograft can technically be classed as a DC, however, in this review they are referred to as separate entities, as corneal xenografts are not always acellular or corneal in origin. In addition, xenograft corneal DCs may have subtle interspecies differences regarding the tissue anatomy and physiology. 

DCs are potentially advantageous in comparison to TE corneal equivalents and KPros because the matrix has the ultrastructure of the native tissue. In addition, many of the inherent biological signals may remain within the matrix [48], which is likely to be important for the maintenance of specific cellular functions and phenotype [41,48]. Such native signals are extremely difficult to synthetically manufacture in TE corneal constructs and are completely lacking in KPros. The three-dimensional (3D) architecture, surface topography and ECM composition all contribute to cell proliferation, differentiation and remodeling processes. The cytokines and growth factors (GFs) present in an intact corneal ECM are potentially powerful modulators of cell behavior [48]. The exogenous addition of growth factors and their effects on cellular and extracellular behavior have been extensively investigated [49,50,51,52,53]. However, the interplay between factors such as dose, binding sites, delivery, sustainability and the ability to control GF activation and deactivation have all made the exogenous use of GFs as a therapeutic agent extremely difficult [48]. These problems can potentially be evaded by the use of DC ECM with intact GFs, proteins and cytokines, providing a scaffold that has all the attendant signals (and inhibitors) in their native, relative amounts [48]. However, it is highly probable that these may be denatured and/or lost during the decellularization process.

It has been estimated that 100,000 corneal transplants are performed annually worldwide [13]. Between 2011 and 2012 in the UK alone, 5871 corneas were reported to be donated to the UK transplant registry [54], of these 3520 were grafted. The remaining corneas were deemed to be unsuitable for transplantation for a variety of reasons: 11% due to medical contraindications; 14% due to insufficient tissue quality; 5% had fungal/bacterial infections; 4% were out-of date and the remaining were incorrectly stored in ethanol. This data for the UK alone represents a possible avenue to source human corneal tissue for decellularization. Recently, the UK Eye Retrieval Scheme (ERS) have issued an upper age limit of 89 years for corneal donation with the aim of improving the quality of donated corneas. This results in a reduction of approximately 7% of corneas into the donor pool, which could also potentially be utilized for decellularization techniques [54]. These numbers represent a potential tissue supply that could be used to produce a standardized bank of human DCs. To realize this, first and foremost, requires the development of appropriate decellularization protocols. Several have been explored in the literature in relation to the cornea.

## 3. Methods of Decellularization

The principal aim of any decellularization technique is the complete removal of cellular material and antigen molecules whilst retaining the structural and functional properties of the ECM [41,55]. The removal of cellular materials and associated debris should diminish any potential host rejection or immunological response. The purpose of corneal decellularization protocols is to produce a functional, biocompatible tissue that is readily transplantable. Maintenance of the tissue architecture, protein and glycosaminoglycan (GAG) content is particularly important to corneal tissue as the corneal stroma has the most organized ECM in the body [56]. It is the uniform collagen fibril alignment of the corneal stroma that is vital to tissue transparency [57,58]. The collagen fibers are a heterogeneous mix of collagen type I (80%) and collagen type V (20%) fibers; collagen type VI is also present, but forms a separate filamentous network that may help negotiate the interactions between the collagen type I and V fibers, and the proteoglycans [59]. The collagen fibrils themselves are weak light scatterers as their diameters (25–35 nm) are less than the wavelength of light with a refractive index close to that of the corneal ground substances [59,60]. They are assembled parallel to each other in 200–250 nm thick orthogonally arranged lamellae. The maintenance of this unique structure is a critical design consideration when planning corneal decellularization protocols. It is important to note that the protocols required to completely remove all cellular material will inevitably cause the most tissue disruption. Likewise, decellularization techniques, which maintain the ECM ultrastructure, are very likely to leave cellular artifacts and residual antigen molecules. Thus, a balance between the ECM ultrastructural disruption and sufficient removal of antigenic and immunogenic material is required. Existing decellularization protocols vary hugely depending upon the tissue, organ and species of tissue being decellularized [42]. There is currently no reliable or standardized protocol for the decellularization of human corneas. Tissue origin (species) and the decellularization and sterilization procedures vary widely amongst studies. It should be noted that the method of preparation of DCs can dramatically alter the cellular and host remodeling response [48]. In addition, published decellularization protocols often lack full characterization of the decellularized tissue, resulting in misleading results. 

Generally, decellularization includes the lysis or removal of the cellular membrane followed by an enzymatic treatment that separates cellular components from the ECM. Cytoplasmic, nuclear components and cell debris are then removed *via* the use of detergents, with mechanical agitation to increase effectiveness [42]. Following the removal of the cellular components it needs to be ensured that all residual chemicals are removed [42]. It may be that the optimal decellularization process requires making use of a number of different chemical, biological, and physical methods to achieve a fully decellularized tissue with minimal damage to the native ultrastructure. Thus far, several techniques have been used on the cornea including biological, chemical, and physical methods (Table 2). Most of these efforts have involved the use of animal-derived tissue, the most common utilizing bovine and porcine corneas [9,61,62,63,64,65,66,67,68,69]. However, feline [70,71] and human tissue [46,72] have been utilized in some instances. An outline of these techniques and their applicability to the human cornea are presented.

**Table 2:** Decellularization methods previously used for the cornea.

Method/Technique	Mechanism of action	Advantages/Disadvantages	References		
**Biological**						
*Enzymatic Agents*						
Trypsin	Hydrolyzes protein and disrupts protein-protein interactions.	Disruptive to collagen structure. Not suited to corneal tissue.	[6,64]		
Dispase	Cleaves peptides associated with basement membrane proteins.	Can aid decellularization process by initially removing epithelium and endothelium. May cause damage to basement membrane.	[6,62,64]		
Phospholiphases A_2_ (PLA_2_)	Hydrolyzes phospholipid components of cells.	No interaction with collagen or proteoglycans.	[68,71,73,74]		
Nucleases (RNase and DNase)	Cleaves nucleic acids and aid in their removal.	Effective at removal of DNA and residual cellular components that have a tendency to adhere to ECM proteins. Incomplete removal of the enzymes may impede recellularization and successful transplantation.	[6,44,64]		
Sera	Serum nucleases degrade DNA and RNA.	Effectively removes cells while maintaining tissue transparency. Use of non-human sera carries risk of cross-species transmission of pathogens.	[12]		
*Non-enzymatic Agents*						
EDTA	Dissociates cells by separating metal ions.	Ineffective at cell removal when used unaccompanied.	[47,69,75]		
**Chemical**						
*Alcohols*						
Ethanol	Dehydrates and lyses cells. Removes lipids from tissues.	Can cause damage to ultrastructure of tissue.	[76]		
Glycerol	Dehydrates and lyses cells. Removes lipids from tissues	Antimicrobial, antifungal, and antiviral properties. Cryoprotectant for long-term tissue storage. Can maintain or restore corneal transparency.	[45,47,64,69,77,78]		
*Acids and Alkalis*						
Peracetic acid	Solubilizes cytoplasmic components of cells. Removes nucleic acids *via* hydrolytic degradation.	Acts to simultaneously sterilize tissue. Poor results in DCs. Can disrupt ECM.	[76]		
Ammonium hydroxide	Hydrolytic degradation of biomolecules.	Can eliminate GFs and reduce mechanical properties.	[46,78]		
*Ionic Detergents*						
Sodium dodecyl sulfate (SDS)	Solubilizes cell membranes and dissociate DNA from protein. Disrupts protein-protein interactions.	Complete removal of cells can be achieved. Can be highly detrimental to ECM structure including disorganization of collagen fibrils and loss of GAGs. Loss of tissue transparency.			[47,61,64,65,69,72,75,76,79]
Sodium deoxycholate (SD)	Solubilizes cell membranes and dissociates DNA from protein. Disrupts protein-protein interactions.	Less effective at removal of cells but can be effective when used with other agents.			[68,79]
*Non-ionic Detergents*						
Triton X-100	Breaks up lipid-lipid and lipid-protein interactions.	Mild and non-denaturing. Less effective than ionic detergent treatments. Can cause damage to ECM structure.	[6,44,46,78,80]		
*Zwitterionic Detergents*						
3-[(3-Cholamidopropyl)dimethylammonio]-1-propanesulfonate (CHAPS)	Has properties of non-ionic and ionic detergents.	Poor cellular removal. Very disruptive to stromal architecture.	[79]		
*Hypo- and Hypertonic Solutions*						
Sodium Chloride (NaCl)	Detaches DNA from proteins.	Remains optically clear. Ability to maintain stromal architecture and retain GAG content. Mixed reports on success of cell removal.	[62,64]		
Tris-HCL	Lyses cells by osmotic shock.	Reduces time required in harsh decellularizing agents.	[6,44,69,75]		
**Physical**						
*Freeze-thawing*	Ice crystal formation causes cell lysis.	Requires subsequent treatment to remove cellular content. Causes pore formation. Disruptions to ECM architecture.	[64,74,79]		
*Hydrostatic Pressure*	Increase in pressure results in cell lysis.	Effectively decellularizes whilst maintaining collagen fibril structure. Kills bacteria and viruses. Expensive.	[63,66]		

### 3.1. Biological Decellularization Techniques

#### 3.1.1. Enzymatic Agents

Enzymatic decellularization protocols are advantageous in that they provide high specificity for the removal of cellular and detrimental ECM elements [41]. However, residual enzymes in decellularized tissues are particularly problematic as they may impair recellularization whilst stimulating immune responses such as severe apoptosis and inflammation, which result in early rejection of DCs [64]. 

Dispase, trypsin and collagenase are commonly used enzymatic treatments. Epithelia and endothelia have been removed following treatment with Dispase II [6,62] before being fully decellularized using a subsequent method. Dispase cleaves peptides associated with specific basement membrane proteins such as collagen IV and fibronectin, but it can also cause damage to the basement membrane if used for prolonged periods [81]. Trypsin is frequently used as an initial or additional treatment to improve the infiltration of other decellularization agents. However, it should be used with caution, as it is disruptive to collagen, despite showing better preservation of proteoglycans [82]. To this end, it may not be well suited to corneal decellularization, where preservation of collagen structure is crucial for the maintenance of the tissue’s optical clarity.

Phospholipase A_2_ (PLA_2_) is an esterase that hydrolyses phospholipid components of cells but does not react with collagens or proteoglycans [68]. When combined with bicarbonate salt solutions it has been shown to effectively remove cells in the cornea while keeping the collagen structure intact [68,71]. However, a significant reduction in GAG content has been noted [68]. The addition of 0.5% sodium deoxycholate (SD) allowed for the incubation time to be reduced from 24 hours to 8 hours by increasing the hydrolytic activity of PLA_2_, thereby substantially reducing loss of hydrosoluble GAGs [68].

Nucleases such as RNase and DNase, are frequently used following enzymatic protocols, to cleave nucleic acids and aid in their removal [44,64,72]. Porcine corneas treated with DNase and RNase resulted in complete removal of cells but the tissue became opaque due to severe distortion of the collagen structure [64]. Transplantation into a rabbit model culminated in immediate corneal melt. From this, it is clear that complete removal of the enzyme is necessary for successful recellularization and clinical use.

Sera, such as fetal bovine serum (FBS), contain nucleases that can degrade both DNA and RNA [83]. As such, it supports the removal of nucleic acid from tissue, but fails to remove immunogenic elements [41]. Various concentrations of FBS have been tested in modified detergent-based decellularization protocols to remove residual DNA fragments [83]. Xenogeneic serum has the disadvantage of introducing immunogenic elements into the ECM which can cause adverse immune responses following recellularization or tissue transplantation into the host [41]. The use of FBS and other sera for complete DNA/RNA removal may limit the time needed in harsh detergents. Moreover, human serum has also been used alone as the decellularizing agent to produce porcine DC, after first mechanically removing the epithelium [12].

#### 3.1.2. Non-Enzymatic Agents

Non-enzymatic treatments include the use of chelating agents and serine protease inhibitors. Chelating agents such as ethylenediaminetetraacetic acid (EDTA) aid cell dissociation *via* the separation of metal ions [84]. However, these same mechanisms can lead to the disruption of protein-protein interactions [41,85]. Chelating agents are often used in combination with enzymes and detergents, as unaccompanied they are insufficient for superficial cell removal [41,86,87,88]. EDTA has been used together with sodium dodecyl sulfate (SDS), an ionic detergent [47], to decellularize corneal tissue [47,69,75]. 

Serine protease inhibitors such as aprotinin, phenylmethylsulfonyl fluoride, and leupeptin, can prevent some of the detrimental effects to the ECM caused by intracellular proteases released by lysing cells. Protease inhibitors are often used as an accompaniment to harsh detergents and decellularizing agents. Commonly used for the cornea is aprotinin [47,61,69,79], an inhibitor of trypsin and related proteolytic enzymes. In these studies, authors have reported minimal damage to the ECM despite the use of harsh decellularizing agents [61,69]. 

### 3.2. Chemical

#### 3.2.1. Acid and Alkali Treatment

Acid and alkali treatments are effective at solubilizing the cytoplasmic components of cells and the removal of nucleic acids [42] by causing or catalyzing hydrolytic degradation of biomolecules [41]. However, such solutions may also remove important molecules such as GAGs from collagenous tissues. For example, acid use has been associated with the damage and removal of collagen from tissues, which reduced ECM strength, but retained sulfated GAGs [41,89]. Likewise, alkali treatments such as ammonium hydroxide have been associated with the removal of GFs and a reduction in ECM mechanical properties [90]. 

Peracetic acid has been investigated as a decellularizing agent [76,91,92]. Limited success has been reported with corneal tissue when used alongside ethanol, however, the authors considered further optimization was necessary [76]. Advantageous for its ability to disinfect, its use has been proposed for preclinical sterilization of acellular scaffolds and reported superior to other clinically accepted methods [93]. In addition, peracetic acid has been reported to retain GAG content and preserve the structure and function of important GFs [42].

Ammonium hydroxide is an alkali treatment that has been used in conjunction with the detergent Triton X-100 to decellularize human corneas [46]. The treatment was reported to result in a complete DC with little apparent effect on the collagen architecture and basement membrane proteins [46].

#### 3.2.2. Alcohols

Alcohols aid decellularization by dehydrating and lysing cells [41,94]. Alcohols such as ethanol and isopropanol are commonly used to remove lipids from tissue. Care should be taken when using them as a decellularizing agent, as they can also act as a tissue fixative [95,96], precipitate proteins [95], damage the ECM ultrastucture [41], and dehydrate the tissues causing tissue opacity [55]. A remedy to this is soaking the tissue in distilled water, which can reverse tissue dehydration [91]. Previously, ethanol treated corneas have resulted in complete tissue decellularization whilst maintaining the overall tissue structure [76]. Interestingly, corneal stromal cells cultured on ethanol treated DCs were reported to proliferate slower and produce more new ECM components, in comparison to those cultured on DCs treated with a detergent method [76]. Moreover, the latter showed a decrease in collagen content over the same culture period.

Glycerine dehydration has been used since the 1960’s for prolonged storage of donor corneas for later transplantation [97,98,99]. More recently, *in vivo* confocal microscopy techniques have shown that antigen presenting cells and stromal cells were, in fact, absent in glycerine-cryopreserved allografts (GCA) used in transplantation procedures into human recipients [45]. These DCs showed promising results when transplanted into patients with high rejection risk due to infection and inflammation [71]. In one such clinical study, no rejection was seen with the GCA, while 10% of eyes that received fresh corneal allografts (FCA) reported episodes of stromal rejection [71]. Another clinical study reported similar findings with no rejection cases in the GCA group and one case of stromal rejection in the FCA group [45]. This suggests that glycerol cryopreservation may be a promising technique for producing DCs for use in corneal grafting procedures, with apparent clinical efficacy. Glycerol has also been used as a post-treatment to restore corneal transparency [47,69] and as a preservation technique for DCs. Detergent-treated bovine DCs snap frozen in 100% glycerol and stored for several days were transparent upon rehydration [78]. Poly(ethylene glycol), an amphiphilic copolymer that damages cells membranes, has been also used as a decellularizing agent [72]; although, it was shown to be ineffective at removal of cells and cellular debris [72].

#### 3.2.3. Detergents

By far the most commonly employed method of decellularization is the use of detergents including, but not restricted to, ionic, non-ionic and zwitterionic detergents. These effectively solubilize the cell membrane and dissociate DNA from proteins [100,101], but at the cost of disrupting and removing valuable ECM proteins [61,90,102]. The removal and disruption of ECM components increases with exposure time, which should therefore be minimized [61].

Non-ionic detergents are considered to be milder than ionic treatments as they target lipid-lipid and lipid-protein interactions, as opposed to protein-protein interactions [55]. Triton X-100 has frequently been used in corneal decellularization protocols [6,44,46,78,79,80]. However, its use has been criticized for the apparent failure to effectively reduce and remove cellular material from tissue [61]. To decellularize the cornea it has been used alone [79,80] and in combination with other agents including hydroxylamine [46,78]; Dispase II, for removal the corneal epithelium [44] and nucleases, to aid in the complete removal of cellular debris [44]. These confounding factors have made it difficult to assess detergent efficiency and the effects on the ECM, in the latter cases. However, in all cases successful decellularization was reported. 

A strong ionic detergent also commonly used in decellularization protocols for the cornea is SDS [47,61,64,65,72,76,79], as it is effective at solubilizing cellular membranes and complete removal of cells has been reported [61,72]. Although more effective than Triton X-100 at removal of cellular material, including complete removal of cell residues, disruption of the ECM has occurred [72] even at concentrations as low as 0.1% (*v/v*) [62]. Contradictory reports have shown that when used at low concentrations (between 0.5 and 1% (*w/v*)), corneal architecture, GAG and collagen content were unaffected [61]. This may be due to inter-species variation or dependent upon the addition of protease inhibitors that can protect the ECM structure and components. Nonetheless, a need for standardized procedures and characterization techniques is highlighted. A reduction in corneal transparency is also associated with the use of SDS [61,62,79]. As previously mentioned, tissue transparency can be restored *via* the use of glycerol [65]. The decellularization process can cause swelling of the tissue, and the dehydrating effects of glycerol return it to a state of deturgescence. Moreover, it has been reported that in a rabbit model, initially opaque DCs recovered their original transparency, 8 weeks post-transplantation, without the use of glycerol treatment [61]. Sodium deoxycholate (SD), another ionic detergent, has also been investigated [71], but appears to be less effective than SDS in the removal of cellular material whilst equally detrimental to the ECM [79]. 

Zwitterionic detergents such as 3-[(3-Cholamidopropyl)dimethylammonio]-1-propanesulfonate (CHAPS) have been criticized as they reportedly leave residual nuclei and cell fragments in the stromal tissue [79]. When used for corneal decellularization, poor cellular removal, as well as damage to ECM proteins has been reported [79].

#### 3.2.4. Hyper- and Hypo-tonic Solutions

Hypotonic solutions, such as tris buffer, can lyse cells *via* osmotic shock, while hypertonic saline can detach DNA from proteins [100]. Decellularization by immersion in alternating hypo- and hypertonic solutions can increase the osmostic effect [41,100,103]. Conveniently, this also supports the removal of cell debris following lysis [41]. The use of hypertonic sodium chloride (NaCl) solution has been investigated for DCs [62,64] and is known to cause minimal damage to stromal architecture and retain many extracellular GFs and proteoglycans. Hence, DCs are reported to be optically clear following treatment [62,64]. However, these treatments are generally less effective at removing cells than, for example, detergent-based methods and incomplete decellularization has been observed in most cases. As a result, protocols may often be modified to include the use of nucleases such as DNase and RNase [6,72]. 

Tissues can be incubated in hypotonic tris-HCl buffers, as a pre-treatment at 4 °C, to initially lyse cells before further treatment with enzymes and/or detergents [6,44,69,75]. This has the advantage of reducing the incubation time needed in the harsher decellularizing solutions. 

### 3.3. Physical Decellularization Techniques

**Figure 1:**
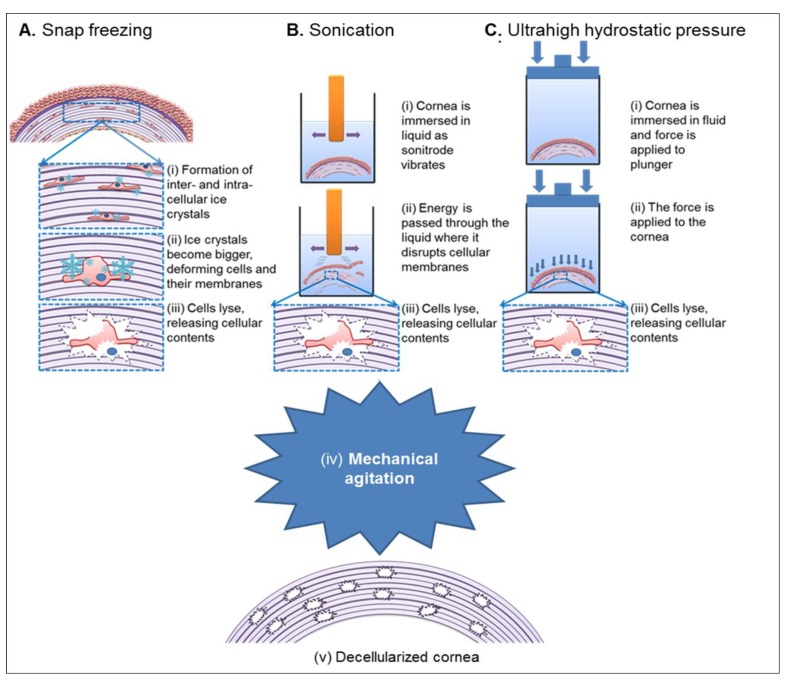
Schematic representation of physical decellularization protocols.

Physical decellularization protocols utilize freezing, pressure, sonication and the use of mechanical agitation [42,55]. Snap freezing of tissues results in cell lysis *via* the formation of intracellular ice crystals that disrupt cellular membranes (Figure 1A). Xaio *et al.* (2001) [74] utilized snap freezing followed by lyophilization to induce pore formation in DCs. The ice crystals formed during pre-freezing are sublimated under vacuum conditions leaving a network of interconnected pores that enabled infiltration by cells [103].

Freezing and incubating the tissue in nitrogen gas has been used to induce apoptosis, as freezing alone can be insufficient [9]. Nitrogen freezing of tissues is a relatively mild treatment when compared to enzymatic or detergent treatments [9]. The application of direct pressure to a tissue can cause cell lysis whilst causing minimal disruption to the ECM [72]. However, both of the aforementioned techniques require additional washing steps to remove the residual lysed cellular material. High hydrostatic pressure (Figure 1C) has been reported to successfully decellularize porcine corneas whilst maintaining collagen fibril matrix and GAG content [63,66]. The use of high hydrostatic pressure is non-cytotoxic, but successfully removes cells whilst destroying bacteria and viruses [55]. One drawback is the cost of this expensive technique, which requires specialized equipment capable of applying pressures of up to 1 GPa to the corneal tissue [55]. Sonication (Figure 1B) and mechanical agitation, in conjunction with chemical and enzymatic treatments, have been used to assist cell lysis and removal [42]. 

Mechanical agitation alone can have the effect of lysing cells, but is more often used to facilitate chemical exposure [41]. Most published decellularization protocols for the cornea involve some form of mechanical agitation for this purpose. The agitation aids the accessibility of the reagents in reaching the cellular and nuclear materials [55] and often orbital or rotating shakers are used. 

## 4. Characterization of Decellularized Corneas

As discussed, decellularization protocols vary considerably in their efficacy [6], resulting in grafts that can elicit a range of *in vivo* effects [41]. Incomplete removal of cellular material can lead to adverse host response, and cytocompatibility issues associated with the DC [104,105,106]. It is therefore important to have sufficient monitoring protocols in practice to discern whether complete decellularization, including removal of all cellular debris has occurred, and ECM integrity has been maintained following processing. Crapo *et al.* (2011) [41] proposed a set of minimal criteria to satisfy the description of “decellularized”: less than 50 ng double stranded DNA (dsDNA) per mg ECM dry weight; less than 200 base pair DNA length; and a lack of visible nuclear material when stained with 4',6-diamidino-2-phenylindole (DAPI) or hemotoxylin. This criteria focuses predominantly on characterizing the removal of DNA, as it has been proven that residual DNA is the cause of the majority of adverse host reactions [104,107]. After assessment of the removal of all cellular components, characterization can then be performed to assess the retention of the native ECM architecture and mechanical properties. Although complete retention of ECM ultrastructure is ideal, minimizing the disruption of the ECM architecture is a more realistic objective when optimizing decellularization protocols.

A standard for characterizing decellularized tissue would be of great significance for translating decellularized products to clinical applications. For the objective of creating ECM products for TE and regenerative medicine purposes, demonstrating successful characterization validates a decellularization technique as effective, consistent, reproducible and suitable for manufacturing purposes. 

### 4.1. Assessment of Removal of Cellular Materials and Retention of ECM Architecture

#### 4.1.1. Removal of Cellular Materials

**Figure 2:**
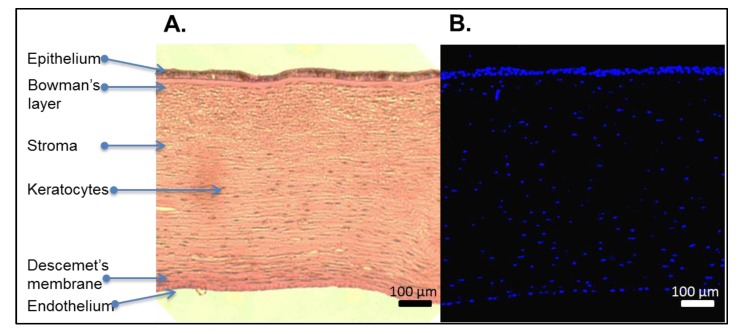
(**A**) Native corneal structure and cellular nuclear staining using hemotoxylin and eosin staining; and (**B**) DAPI.

The most popular method of assessing the removal of cellular material is performance of a DNA stain, followed by imaging. Amongst possible stains, hemotoxylin is used most routinely to assess the degree of decellularization, and is usually performed alongside eosin (Figure 2A) to assess basic ECM architecture of DCs [9,46,62,63,66,67,68,69,72,78,108,109]. Other commonly used nuclear stains include fluorescent DAPI [61,62,72] (Figure 2B), Hoechst [9,68] and propidium iodide [72]. Cell apoptosis that occurs during the decellularization process, can be assessed by assays such as the TUNEL assay, which detects levels of DNA fragmentation, by fluorescently labeling the terminal end of nucleic acids [9,67]. Although, DNA staining and imaging is the standard for assessing decellularization, and is used throughout the body of literature, it is relatively insensitive and offers no quantitative information. 

For quantitative information regarding residual DNA, DNA can be extracted from the DCs and spectrophotomic assays such as Pico green or Hoechst used [83,90]. Remaining DNA fragment size can also be assessed by gel electrophoresis, to determine how successful the decellularization process has been in breaking down the cellular components. The disadvantage of performing these assays and staining protocols to assess residual DNA, is that destruction of the sample is required. 

#### 4.1.2. Biological Assessment of ECM Architecture

Preservation of the native tissue architecture and ECM composition during tissue decellularization is the ultimate aim of decellularization protocols [41]. This is true of all decellularized tissues, but is of particular interest to DCs, as the corneal stroma has the most organized ECM in the body and it is this structure that is responsible for corneal transparency [56]. Basic histology can be used to compare the architecture of DCs with the native cornea. Stains such as eosin and van Gieson’s have shown immediate changes in collagen structure [61,79]. However, basic histology does not always offer enough specificity, and in such cases, immunohistochemistry is a useful tool as it is capable of detecting corneal specific ECM proteins. Corneal specific proteins commonly utilized include collagens I, II, III, IV and V, keratin, fibronectin and laminin, present in the basement membrane [46,61,72]. If a fully intact DC is the final goal, then determining the presence and integrity of the Bowman’s layer and Descemet’s membrane is important, and the identification of specific proteins, rather than a general eosin stain, will give more information. To identify other components of the DC, stains such as Alcian blue have been used as a way of assessing whether the GAG content within the corneal stroma has been retained [62,90]. 

#### 4.1.3. Toxicity and Immunogenicity of Decellularized Corneas

It is important to consider how the host may respond to a DC, specifically the immune response and particularly when employing xenogeneic decellularized tissues. An important factor in the rejection of xenogeneic tissue is the α-Gal (Galα1-3Galβ1-4GlcNAc-R) epitope [110,111,112]. Ideally, traces of this epitope should be removed throughout the decellularization process, evidence for which has been provided by immunohistochemistry or enzyme-linked immunosorbence assays (ELISA) [62,113]. The presence of the α-Gal epitope is one potential driver to move away from the use of xenogeneic grafts.

The use of allogeneic human tissue may also produce an immune response through the presence of major histocompatibility complex (MHC) antigens displayed on donor cells; specifically in humans referred to as human leukocyte antigens (HLA). There are two classes of HLA antigens, class I, which are expressed by almost all nucleated cells of the body including cells of the cornea; and class II, which are only expressed by antigen-presenting cells such as macrophages, B cells and dendritic cells, not normally present in the cornea. HLA class I antigens present peptides from inside a cell. The T-cells and natural killer (NK) cells of a host recognize foreign HLA class I antigens, such as those from allogeneic implanted cells, and act to destroy the cells, initiating the immune response and inflammation, resulting in graft rejection [114,115]. However, DCs should not cause an HLA-mediated immune response because in theory, all cells and cellular debris, including HLA antigens, have been removed during the decellularization process. 

Performing transplantations of DCs *in vivo* into animal models provides crude information on the relative immunogenicity of the implant [6,9,63]. *In vivo* models, typically rabbit, are used to determine the biocompatibility of DCs. This has often been assessed through, recruitment of immune competent cells, stromal cell infiltration, epithelialization, transparency and clinical assessments of pathological vascularization, and signs of rejection. Often the first and easiest assessment of immunogenicity of decellularized grafts is to implant the DC subcutaneously *in vivo* [73]. As of yet, there have been no studies investigating the systemic immune response caused by implantation of a DC.

*In vivo* animal models also give an impression of degradation rate of DCs. DCs are not designed to degrade *in vivo*, as many TE constructs do, but are supposed to be remodeled by cells to integrate into the existing tissue. If degradation occurs it is likely to be related to the immunogenicity of the implant. Wu *et al.* (2009) [68] report no degradation and no signs of inflammatory cell infiltration within their implanted PLA_2_ decellularized constructs within 12 months of implantation [68]. However, in transplanting an *ex-vivo* recellularized DCs, Zhang *et al.* (2007) [6] noted complete degradation within 12 weeks. Importantly this was associated with an acute infiltration of inflammatory cells. Many groups choose to assess cytotoxicity of the DC *in vitro* by recellularizing the DC with the various cell types present in the cornea and performing viability assays, such as MTT [6]. Extract cytotoxicity assays are often performed to assess the effect of leachables on cells. An assessment of the safety of both extractables and leachables is a requirement of the Food and Drug Administration (FDA) for medical device submission [116]. Scaffolds are placed in medium for a defined period of time and the effect of the extract on the cytotoxicity of epithelial cells or stromal cells is measured by through the use of viability assays [6,12,67].

### 4.2. Imaging of Structural Architecture and Transparency

**Figure 3:**
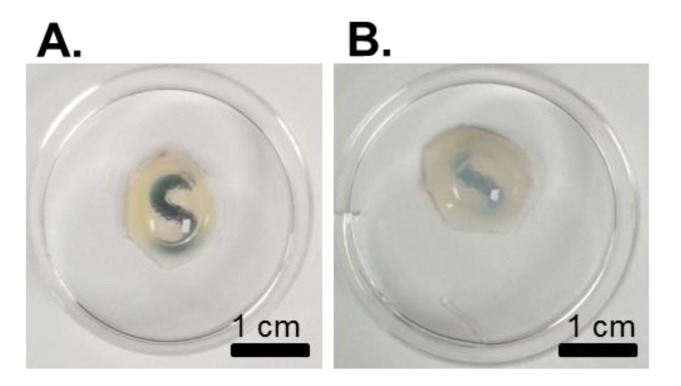
Basic macroscopic evaluation of decellularized corneas can provide a crude marker of the success of a decellularization protocol; comparisons of (**A**) a non-treated cornea; *versus* (**B**) a cornea treated with strong ionic detergents. It is apparent that the detergent-treated cornea is opaque in appearance when compared to the transparent non-treated cornea. These opacities are caused by disruption of the tissue architecture due to the decellularization process.

Although histological and immunological procedures are useful techniques in biomedical science, fixation, labeling and processing techniques can distort cellular expression and tissue architecture [117], are often destructive, and only provide information on very small areas of the cornea [118]. Thus, the need for non-destructive, high contrast, high resolution and large area imaging techniques is apparent [117]. Initially, macroscopic evaluation of the tissue can be performed, as disruption to the tissue architecture can result in the occurrence of opacities (Figure 3). However, this crude mechanism may not be sufficient to determine micro-scale changes. Furthermore, the transparent nature of corneal tissue often makes imaging of corneal internal structures challenging. The following sections discuss existing and relevant techniques that have been successful and show potential in monitoring of corneal tissues. This may be particularly useful to the decellularization process in assessing the reproducibility and the introduction of “quality control” measures to determine what classes as a successful or acceptable decellularization protocol. 

#### 4.2.1. Light Microscopy Techniques

Light microscopy is a relatively simple technique used in the earliest qualitative studies of corneal architecture, leading to the first descriptions of the stromal lamellar structure [119]. Transverse tissue sections are taken and immunostaining of samples allows for the distributions of different types of collagen to be determined [119]. However, the processing and sectioning protocols required to visualize the microscopic structures may cause shrinkage or distortion of the tissue samples [119]. Additionally, the light reflected and scattered from the structures surrounding the point of interest can obscure the image outside of the focal plane, reducing resolving power and image content [120,121]. Many techniques are available that modify the light path to improve the resolving power and contrast of specimens. Phase contrast microscopy, is one of the simplest techniques and is most useful in revealing cellular structures that cannot be seen with standard light microscopy.

Polarized light microscopy allows quantitative evaluations of collagen organization [119,122]. Colored polarized images provide an appreciation of the different orientations of the collagen fibrils in normal and pathological corneas. This technique shows promise in discerning structural changes in collagen that may have occurred during decellularization. 

Confocal microcopy allows for detailed *in vivo* observations to be made [119], allowing for lateral corneal structures such as the Bowman’s membrane and stroma to be visualized at a resolution of 1–2 µm [120,123], with a depth of field ranging from 10 to 26 µm [121]. Current confocal systems are frequently used in both laboratory and clinical settings [123]. They are utilized for *in vivo* ophthalmologic observations for disease diagnosis [117], degenerative disorders, the effects of refractive surgery procedures [120,121] and the effect of contact lens wear [123] as they can detect stromal deposits and stromal matrix disruption on living tissues *en face* [121], without the need for tissue fixation [120], invasive preparations or staining protocols [124]. Confocal microscopy can also be used to measure corneal transparency by measuring the intensity of backscattered light relative to a healthy cornea. This may make a good technique for monitoring DCs compared to healthy, native tissue. 

Light microscopy techniques are valuable for initial analysis of surface corneal structure. However, information regarding the ECM quality following decellularization can only feasibly be achieved *via* destructive sectioning techniques, so although useful during the development of appropriate decellularization techniques, it is not applicable as a screening tool.

#### 4.2.2. Electron Microscopy

Electron microscopy is a predominantly qualitative technique used to provide localized information on stromal architecture [125]. Compared to light microscopes, electron microscopes have greater resolving powers to reveal detailed ultrastructures.

Transmission electron microscopy (TEM) is capable of revealing ultrastructural details of corneal tissue such as fibril diameter, interfibrillar spacing and order of collagen fibrils and is a central technique to the understanding of the corneal architecture [57,119,126,127]. Specialized staining techniques and metal labeling in conjunction with TEM can reveal the location and distribution of binding sites of proteoglycans, that are vital to collagen organization [119]. TEM has previously been used to compare species differences in corneal structure [128] and is able to detect and measure the thickness of the Bowman’s and Descemet’s membranes [128]. However, it should be noted that the fixing protocols and embedding resins required for TEM can alter the collagen fibril diameter and intrafibrillar spacing in the corneal tissue [127]. Thus, this may distort or alter the apparent effect that a decellularization technique has on a tissue and needs to be considered during analysis. 

**Figure 4:**
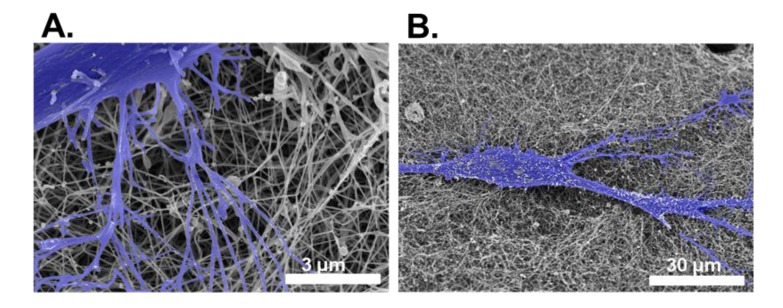
False-colored images of corneal stromal cells (blue) and collagen structure in tissue-engineered corneal stromal constructs at relatively (**A**) high; and (**B**) low magnification imaged using SEM.

Scanning electron microscopy (SEM) is a technique that probes the surface of the specimen providing information regarding the surface and topography of the sample. Although the resolving power is less than TEM, SEM has been utilized for the evaluation of surface and cellular morphology of the anterior surface of the cornea and the determination of 3D lamellar structure [119], in both native and regenerated tissues (Figure 4). It can also be used to image larger areas than TEM, as it is not reliant upon transmission. Unfortunately, critical point drying techniques used to prepare specimens for SEM are associated with a variable amount of specimen shrinkage [129], and harsh fixatives may be required. Concerning evaluating decellularized corneal tissue, a combination of both SEM and TEM has been successfully used [72].

#### 4.2.3. Second Harmonic Imaging

In cornea, second harmonic imaging allows for high-spatial resolution and contrast images of corneal structures to be achieved that are comparable to light and electron microscope studies [130]. The increased resolution allows for deep imaging of corneal depths of up to hundreds of microns to be investigated [131,132], displaying the 3D interwoven lamellar structures and the Bowman’s layer [130]. This non-invasive technique negates the need for staining, sectioning and additional processing of the tissue [130,131].

#### 4.2.4. High Frequency Ultrasound

High frequency ultrasound utilizes acoustic reflections and scattering to create high resolution, near-microscope, quantitative imaging of the cornea [133,134]. High frequency ultrasound (50 MHz) allows for resolving powers up to 30 µm and improved tissue differentiation, in comparison to conventional ultrasonic techniques (8–10 MHz) [134]. The technique has been utilized in many areas concerning corneal tissue including: quantitative assessment and analysis of corneal epithelial thickness; pathology in response to chronic exposure to various drugs [135]; measurement of the depth of incisions following radial keratoctotomy [133]; and most importantly and applicable to screening of DCs, stromal thickness measurements and non-invasive assessment and comparison between normal and scarred corneal tissue [134,136]. It is an advantageous technique as it is non-destructive [135], does not require prior staining or sectioning of the sample, and can provide information on the corneal tissue microstructure continually over areas up to 10 mm [134,136].

#### 4.2.5. Optical Coherence Tomography

Optical coherence tomography (OCT) and ultrahigh resolution OCT are both non-invasive imaging techniques with micrometer resolution, capable of measuring the cross-sectional structure and thickness of materials up to 2 mm [137,138]. Commercially available OCT systems are readily used clinically [139]; frequently by ophthalmologists to examine the ocular structure, particularly the retinal structure. The resolution of clinically used OCT systems is often equivalent to a low-powered light microscope. Confocal and multi-photon techniques are capable of imaging up to 200 μm depth and OCT is advantageous in that it does not require fluorescent labeling of samples [139,140] and is relatively inexpensive and easily set up, thus is a suitable technique for monitoring DCs. OCT is clinically relevant [138] because it allows for fast, sterile, non-destructive, *in situ*, real-time investigations [138,140]. Presently, the resolution of OCT technologies currently used in clinical settings is significantly below what is theoretically possible. Ultrahigh resolution OCT has the potential to allow for the visualization of intra-corneal architectural morphology with axial resolution of 2–3 µm [141], capable of distinguishing the Bowman’s layer and stromal morphology [137], which would be a beneficial way of rapidly characterizing corneal tissue following decellularization. 

#### 4.2.6. X-Ray

David Maurice first pioneered the use of X-rays to examine the collagen fibril arrangement of corneal tissue in 1957 [57,142]. Subsequently, X-ray scattering has been extensively used to provide information regarding the typical structural characteristics of the cornea [58,125,143,144] including the determination and quantification of the orientation and content of the collagen fibrils throughout the entire cornea and limbus [57,118,143]. X-ray scattering has been used to establish whole corneal thickness [125] and differences in collagen fibril orientation of normal, keratoconus corneas [143] and aging corneas [144]. Theoretically, this technique could be used to determine differences in normal corneas and DCs, as the scattering patterns can detect changes in the collagen fibril arrangement [143]. Advantageously, X-ray scattering techniques do not require tissue processing that could potentially disturb the corneal structure [125]. 

#### 4.2.7. Atomic Force Microscopy

Atomic force microscopy (AFM) has been utilized to investigate the corneal stroma and sclera, and is frequently used to confirm TEM observations [119]. However, unlike TEM and SEM, it is capable of measuring the “gap-zones” between the corneal collagen fibrils [145]. AFM involves scanning the corneal surface with a sharp, finely pointed tip controlled by a piezoelectric motor [119,145]. The undulations of the tip are monitored with a laser-diode detector and translated into a 3D topographical image [119,145]. AFM is advantageous in that measurements can be taken in both vacuum and non-vacuum (air or liquid) environments and it does not require metal-coated conductive samples, as are required in electron microscopy [145]. Thus, AFM could be used as a non-destructive screening tool for DC matrices.

### 4.3. Characterization of Mechanical Properties

Ultimately, decellularization protocols aim to remove all cellular and nuclear material from the tissue. It is important to be able to evaluate the ability of protocols to decellularize the corneal stroma without reducing tissue transparency or mechanical strength [9]. Changes to the 3D architecture of the tissue due to decellularization protocols will result in alterations to the biomechanical properties of the tissue. Thus, the maintenance of tissue strength and elasticity can be used as a measurement of the preservation of the ECM. *In vivo*, the cornea is a viscoelastic [146,147], anisotropic [16], load bearing tissue, constantly subjected to forces from intraocular pressure and the movement of the eyelid. Thus, it can be assumed that these forces will have an effect and are affected by cell and tissue behavior. For example, the biomechanical properties of the cornea have been shown to be an important component of the final refractive effect [148]. The aforementioned characterization techniques are primarily concerned with structural and biological alterations to the ECM. To date, there are no studies that investigate the effect that decellularization has on the viscoelastic properties of the tissue [55]. The following section summarizes techniques that have been previously used and adapted, to characterize the mechanical properties of the cornea and equivalent tissues. However, many mechanical tests used with corneal tissue and equivalents, such as decellularization protocols are not specific to corneas; they are existing tests that have been adapted for use on cornea. As such, the various techniques suffer from disadvantages and may need further optimization. A summary of some of these methods and their advantages and disadvantages can be found in Table 3.

Measuring mechanical properties of biological constructs is typically very challenging. The measurement of mechanical properties under sterile conditions is also highly desirable when monitoring biomechanical processes. Although many mechanical testing techniques are available, it is difficult to compare and contrast information, as there are considerable differences in the technological means of carrying out the tests. Additionally, the interpretation of results can also contribute to discrepancies between data acquired by mechanical testing [149].

**Table 3:** Tests used to monitor the mechanical properties of corneal tissue and tissue equivalents; a brief description and common applications; advantages and disadvantages.

Method/technique	Description/applications	Advantages	Disadvantages
**Bulge/inflation testing**	Involves inflation of the whole tissue/membrane/film through a window in the substrate and measuring the displacement as a function of the applied pressure [146,146,150]. Used to measure mechanical strength of thin films, membranes and corneal tissue. Can determine constitutive relationships of corneal tissue [151].	No gripping problems. Maintains corneal integrity [148]. Reliable technique. Enables intrinsic properties on a layer-by-layer basis to be determined [152]. Can be used to simulate intraocular pressure [148]. Can be performed under physiological conditions [148,153]. Whole tissues can be measured. Previously used to characterize DCs [55] and the biomechanical stability of xeno-tissues for human transplantation [148].	Complex procedure [152]. Difficulties in controlling the applied pressure; *i.e*., leaking or trapping of dissolved air. Most inflation tests do not account for corneal anisotropy [148], inhomogeneity or viscoelasticity [153].
**Compression testing**	Test materials are compressed between two plates and deformed under a known load. Used to determine the mechanical behavior of materials under crushing loads [154,155].	Regularly used in TE applications [156]. Confined and unconfined tests can be performed. Gives a comprehensive evaluation of a materials load-bearing capacity [155].	Does not account for corneal curvature. Involves flattening of the tissue. Difficulties associated with applying pressure evenly. Destructive [157].
**Holographic interferometry**	Uses laser light to create an image. Can be used to compare pressure changes in healthy and diseased corneas [158]. Previously used to determine differences between intact, incised [159] and laser ablated [160] corneas. Measures the elastic modulus [161] and extensibility of *in vivo* corneas [162].	Very sensitive, precise method. Allows for direct comparison of two adjacent areas in a single sample. Non-destructive. Allows for repeated measures of a sample [158].	Rarely used by researchers. Limited to use in linear elastic materials under small deformation [146].
**Indentation testing**	A well-defined indenter is used to deform test materials and measure their force-displacement curves; this can be used to calculate the elastic modulus. Traditionally used to measure the hardness of materials.	Can be adapted to be non-destructive. Can be adapted to test for prolonged culture periods under sterile conditions [146,163]. Fast, online real-time measurements. Can be performed on a nanometric scale. Suspending the materials eliminates problems associated with backing substrates.	Cannot be used to test high stiffness materials.
***In vivo* mechanical testing**	Pulses of air or poking mechanisms are used to test materials. Used to measure corneal hysteresis by comparing inward and outward pressure values [164].	Can be performed on live patients. Changes in mechanical properties can be directly linked to medical conditions [165].	*In vivo* tests are difficult to apply to *in vitro* models. Unsuitable for prolonged culture periods. Sample contamination. Creep, stress-relaxation and stress-strain relationships are yet to be assessed.
**Strip extensiometry (coupon testing)**	Involves applying a tensile force to dissected strips with constant width of corneal tissue that are gripped and stretched *via* the application of a tensile force. Is used to calculate the Young’s modulus, yield strength and ultimate tensile strength of the cornea and equivalents.	A relatively simple technique [151]. Inexpensive. Can be used to compare corneas of different species with each other [152,166]. Commonly used to determine the properties of engineering materials [151]. Has been previously used to characterize DCs [55]	Unreliable [151]. Does not account for corneal curvature unless complex calculations are employed [162]. Stress distribution of corneal tissue is not uniform. Destructive [151]. Cannot be used to study whole tissues. Problems associated with sample gripping. Complex calculations involved [151].
**Ultrasound**	A biomicroscopy technique which utilizes high frequency transducers, creating 2D images from backscattered ultrasonic waves [167]. Used to visualize numerous ocular structures and to detect *in vivo* foreign bodies.	Allows for detailed surface imaging up to 5 mm in depth. Allows for quantitative assessments of the anterior ocular surface to be made [168]. Non-invasive technique. Can be applied *in vivo* and *in vitro.*	Expensive. Yields results that are too high when compared to known measurements [168].

## 5. Recellularization Techniques

For DCs to have clinical utility as a suitable alternative graft material it is critical that they perform as well as or better than cadaveric donor corneas *in vivo*. It is therefore important that the processed grafts can integrate into the host tissue, and form *functional* corneal tissue. Restoration of function in DCs is likely to be highly reliant on the grafts ability to be recellularized by the relevant cell types of the tissue. Recellularization strategies for DCs can be divided into two areas: (i) *In vivo* implantation of the DC, allowing host cells to repopulate the graft post-surgery; or (ii) Seeding the construct *ex vivo*, for downstream transplantation of a cellularized graft. 

### 5.1. *In Vivo* Recellularization

The clinical use of a DC, relying on host cell repopulation to create the desired functional tissue, is arguably the simplest and lowest risk strategy. Clinical delivery of a tissue seeded with even one cell type significantly raises both the manufacturing complexity, as well as the clinical evidence threshold required by regulators [169]. 

The majority of research into *in vivo* recellularization has been reported in animal models. As discussed earlier, much of this work has explored recellularization through investigation of the biocompatibility of such constructs in a xenotransplantation model, typically involving grafting porcine DCs into healthy and offended corneas of New Zealand white (NZW) rabbits. The surgical delivery of these constructs has been performed by one of two procedures: a small disc of the DC inserted into a small pocket within the host corneal stroma (intra-lamellar grafting) or alternatively, a partial thickness corneal transplant (anterior lamellar grafting).

#### 5.1.1. Intra-Lamellar Grafting

**Figure 5:**
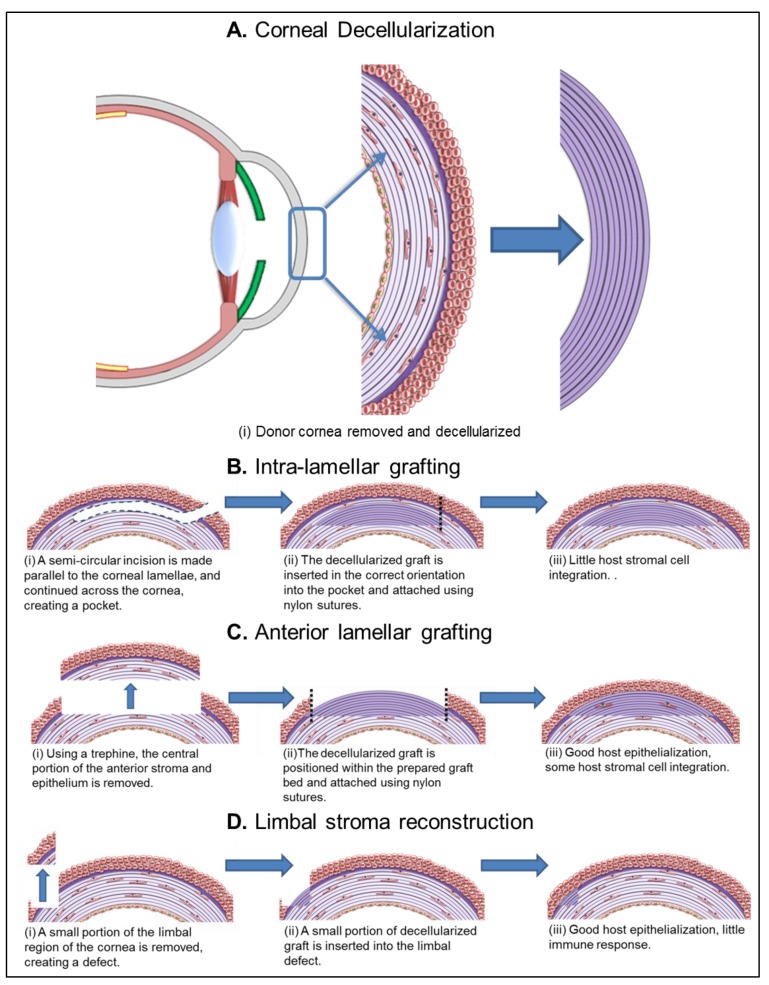
Schematic representation of *in vivo* recellularization techniques.

The most common surgical model used to assess corneal substitutes is implantation of the substrate within intra-lamellal pockets in rabbit corneas [44,63,65,66]. Typically, the host corneas have not had prior pathologies and are not modeling disease states. A small incision is made on one side of the host cornea, parallel to the corneal surface, forming a stromal pocket (Figure 5Bi). A test substrate can easily be inserted within, before the corneal pocket is sutured closed (Figure 5Bii). Inserting a DC disc into the corneal stroma offers little understanding about the quality of epithelial and endothelial integration, although the method does demonstrate stromal cell migration. This model is most useful as an assessment of graft immunogenicity. Histological cross sections at the experimental endpoint can be used to show if undesirable mononuclear immune cells interact with the investigated matrices.

The range of techniques used to decellularize corneas has resulted in variable rates of success of stromal cell infiltration. Interestingly, histological analysis of DCs produced using high hydrostatic pressure showed little host stromal cell infiltration even after periods of 1 year [63,66]. The same observations were also reported by Xu *et al.* (2008) [44] and Pang *et al.* (2010) [65], when grafting acellular corneas processed with Triton X-100 and SDS respectively. Despite this, these acellular grafts were not seen to ellicit an immune or inflammatory response in the form of an influx of immune competent cells. Nor were they seen to produce any undesirable responses by clinical examination [44,63,65,66].

DCs which can be naturally repopulated with corneal stromal cells, without eliciting an inflammatory response *in vivo*, have been a target for several groups [61]. Du and Wu (2011) [61] reported stromal cell infiltration in SDS treated grafts up to 24 weeks after grafting. However, close analysis of the histological cross sectioning appears to give limited evidence of stromal cell in-growth. The most positive results demonstrating stromal cell in-growth using this model have been reported by Xiao *et al.* [74] (2011). In this investigation, the cornea, decellularized by PLA_2_, was freeze dried to improve the porosity of the stromal matrix. Ninety days after intra-lamellal implantation, a large number of stromal cells were seen to have repopulated the implant. 

#### 5.1.2. Anterior Lamellar Grafting

Another popular alternative to intralamellar grafting is a partial thickness graft, also known as anterior lamellar grafting. This model involves prior preparation of a graft bed through removal of a 6 mm diameter, 100 µm deep, portion of the host cornea, removing the epithelium and anterior stroma (Figure 5Ci). DC matrices are then grafted to the bed with the use of nylon sutures (Figure 5Cii). This model is far more useful from a translational perspective than that of intra-lamellar grafting. Firstly, the use of partial thickness corneal grafts is becoming routine clinical practice in treating indications in which only the epithelium and anterior stroma is damaged [5,170]. Secondly, this method allows assessment of surface epithelialization of the graft materials, in addition to stromal cell in-growth [68].

Using anterior lamellar grafting, Wu *et al.* (2009) [68] were able to demonstrate excellent epithelialization and stromal cell infiltration in PLA_2_ treated corneas. It was reported that bi-layered epithelialization of the graft was achieved within 10 days of implantation with further stratification occurring over time. Li *et al.* (2011) [71] reported more recently, complete re-epithelialization of feline grafts decellularized with PLA_2_ over 4 days, although the surgical delivery in this report was more attuned to a full thickness corneal transplant. Other groups have also described a full epithelial monolayer formed over the DCs within a 4 to 10 day timeframe [64,69]. Wu *et al.* (2009) [68] noted an increase in transparency of DCs over time, attributed to the developing epithelium, which was able to regulate the stromal hydration. On implantation, the PLA_2_ treated DCs swelled considerably, with notable changes to the collagen fibril diameter and arrangement. However, post-epithelialization of the graft, the native character of the stromal fibrils was restored through the gradual reduction in water content, in turn leading to improved transparency [171]. The same report also acknowledged significant levels of migration of “activated” or fibroblastic stromal cells from the host stroma into the implant. The numbers of these cells within the grafts were seen to change over time, with cells in the graft after 80 days reported as quiescent, a phenotype akin to keratocytes. However, it is important to note that these phenotypes were characterized by morphology alone, in TEM experiments. 

There have been reports of DCs delivered by anterior lamellar grafting causing adverse reactions. The grafting of porcine corneas decellularized through a hypotonic treatment was seen to recruit huge numbers of cells into the matrix [69]. Whilst the authors gave no evidence as to whether these were indeed CD4 or CD8 positive immune competent cells, it was acknowledged that cell number and position within the graft would normally be indicative of an immune response. Despite this, the authors claim to have seen no evidence of rejection through clinical examination.

#### 5.1.3. Limbal Stroma Reconstruction

There is only one example of the *in vivo* use of DCs in limbal reconstruction. Herein, Huang *et al.* (2011) [73] assessed several different constructs as scaffolds suitable for limbal stroma reconstruction. This *in vivo* model involved the excision of a small portion of sclero-corneal lamellae creating a limbal defect (Figure 5Di). An anterior lamellar graft of one of four constructs was then used to assess suitability in limbal reconstruction. The study used PLA_2_ to decellularize a portion of limbal stroma, and investigate how well this scaffold could restore a limbal defect. The decellularized limbal stroma afforded the best results of the four substrates tested, showing the least infiltration by immune competent cells. In addition, the decellularized limbal stroma cultivated the most desirable cellular phenotype of epithelial cells, repopulating the acellular graft. This paper was an important addition to previous work that had demonstrated the utility of PLA_2_ treated corneal grafts [68,74].

### 5.2. *Ex Vivo* Recellularization and Cell Sources

An alternative assessment of recellularization of DCs is seeding individual cell types upon or within the acellular matrix *ex vivo*. These cellular constructs are then typically cultivated in tissue culture conditions relevant to the cell source under investigation. *Ex vivo* recellularization experiments have been used to either assess the biocompatibility of the matrix, or to develop a cellularized graft, which would be suitable for transplantation [6,65]. As would be expected there have been reports of the use of epithelial, stromal and endothelial cells. Sources of disagreement between protocols in this area appear to be variations in cell source, seeding density, cell delivery method, and culture conditions. 

#### 5.2.1. Epithelial Cells

*In vivo*, there are several layers to the corneal epithelium and cells are constantly being renewed *via* a balanced process of proliferation, differentiation and cell death. The original source of these epithelial cells is the limbal epithelial crypts, a stem cell niche found in the outer ring of the cornea [172,173]. As the epithelium is self-renewing, as long as the limbus of the cornea is intact, an ideal replacement matrix would allow for re-epithelialization by the host’s own cells. If a limbal stem cell deficiency is present or the limbus is missing, a limbal stem cell transplant can be performed [174]. It is possible to culture human corneal epithelial cells *in vitro*, either by explant or single cell cultures [175,176,177,178,179], and it is conceivable that cultured epithelial cells could be used to repopulate the epithelial layer of a DC, but there would need to be an intact basement membrane to support the cells. There is also the possibility of using stem cells, such as induced pluripotent stem cells (iPSC) [180,181], embryonic stem cells (ESCs) [182,183,184] or adult stem cells to generate corneal epithelial cells for implants [185,186,187].

**Figure 6:**
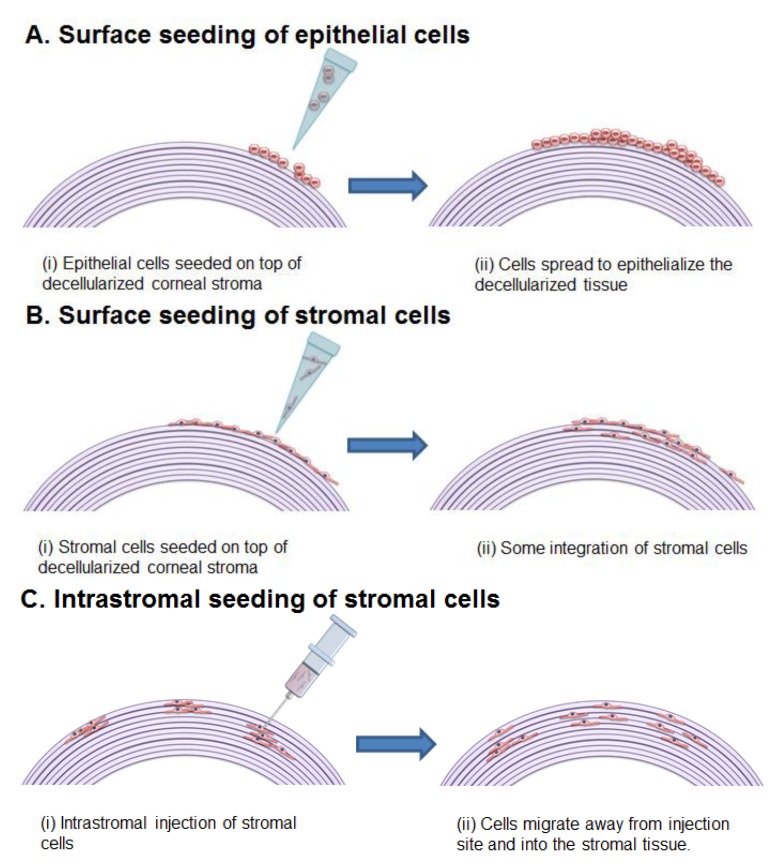
Schematic representation of *ex vivo* recellularization techniques.

Multiple groups have investigated the seeding of epithelial cells on the surface of DCs. The standard seeding method is to pipette extracted primary limbal epithelial cells, usually of rabbit or human origin, onto the denuded basement membrane [44,65,67,69,72,79] (Figure 6A). An area of contention is the optimal seeding density for these cells. Seeding densities have been reported as 5 × 10^3^ cells/mm^2^ [44,79]; 1 × 10^4^ cell/mm^2^ [69]; and ~1.5 × 10^4^ cell/mm^2^ [72]. Despite these differences in seeding density, all groups have demonstrated a stable stratified or bilayered epithelium across the surface of the DCs [44,69,72,79].

#### 5.2.2. Corneal Stromal Cells

The stroma of the cornea contains a population of cells conventionally known as keratocytes [188,189]. *In vivo*,keratocytes sparsely populate the stroma, remaining quiescent and exhibiting a dendritic morphology, with extensive cellular contacts [190,191,192,193]. The function of these cells is to maintain structure and transparency of the stroma by producing and maintaining ECM proteins, such as collagen and proteoglycans [58,190,194,195,196,197,198,199,200]. Markers traditionally used to identify keratocytes include aldehyde dehydrogenase (ALDH), keratocan, transketolase, CD133 and CD34 [191,201,202,203,204,205]

Recellularization of the stroma is arguably the most challenging, from a TE perspective. This is because the cells are required to be in an even dispersion in a dense 3D matrix. In addition, keratocytes in the native healthy corneal stroma are quiescent and thus do not proliferate [206]. For recellularizing the corneal stroma, groups have predominantly used primary stromal cells either directly following extraction from the cornea [76] or extracted and expanded before seeding [6]. These cell types have then been delivered to the DC in an attempt to repopulate the stroma.

Keratocytes can be isolated from the corneal stroma using collagenase treatment and subsequently cultured *in vitro*. However, once transferred to tissue culture plastic, the keratocyte phenotype rapidly disappears and alternative cell populations emerge, dependent on the culture environment [207,208,209]. When grown in high serum-containing medium, such as Dulbecco’s modified Eagle’s medium (DMEM) with 10% fetal bovine serum (FBS), the extracted stromal cells take on a fibroblastic phenotype, and are said to be “activated” [207,210,211,212,213,214]. *In vivo*, this “activation” is associated with response to injury, as the keratocytes begin to exhibit morphological characteristics of fibroblasts and commence tissue remodeling [207,215]. In severe injuries or later stages of remodeling, a myofibroblast phenotype also appears, that actively secretes ECM components, such as α-smooth muscle actin (α-SMA). This can cause scar formation and loss of corneal transparency [190,216,217]. This transition *in vitro* is useful when modeling corneal injury, but for the purposes of recellularizing DCs, this is no longer the native keratocyte phenotype that is required. Previously, it was thought that once keratocytes became fibroblastic, it was an irreversible state, but more recently there is evidence that *in vitro* restoration of the native niche environment and careful tailoring of culture conditions has the potential to revert cultured (activated, fibroblastic) stromal cells back to a healthy, native keratocyte phenotype [218,219,220]. A 3D-environment may be an important factor in instigating this reversal, thus recellularization of DCs with activated stromal fibroblasts, could cause cells to revert to their quiescent keratocyte phenotype.

Evidence has been presented demonstrating that the corneal stroma contains progenitor mesenchymal stem cells (MSCs) that play a role in corneal regeneration. These cells exhibit properties of MSCs, expressing MSC-associated cell surface markers such as CD29, CD73 CD90 and CD105, and possess the ability to differentiate down the osteogenic, chondrogenic and adipogenic lineages [191,221,222]. Recently, our group has discovered several sub-populations of different phenotypes within corneal stromal cells, including MSCs and the characteristic keratocyte, which possess the CD34^+^ phenotype. We have shown that these CD34^+^ cells have the ability to differentiate into corneal epithelial cells (data submitted for publication) and it may be possible that these stem cells play a role in corneal regeneration *in vivo*. Therefore, corneal stem cells may have the potential to be exploited for recellularization of DCs. 

Many groups have seeded stromal cells on the anterior surface of DCs (Figure 6B), expecting gravity and cell migration to afford a good distribution of cells in the stroma [6,44,62,76]. The principal limitation with this seeding method is inadequate cell infiltration. The stromal cells form confluent layers on the anterior surface and show little sign of in-growth [44,76].

Alternative approaches to improve the distribution of stromal cells in the matrix have also been reported using an intra-stromal injection into the lamellae with needles of small bore size (Figure 6C) [65,69,72,74]. Typically, five to ten injections are made at different positions into the corneal stroma. Seeding densities reported for intra stromal injections have been reported as 5 × 10^4^ cells/stroma [74]; 5 × 10^5^ cells/stroma [65]; up to ~ 1.4 × 10^6^ cells/stroma [72]. Shafiq *et al*. (2012) [72] delivered the largest number of stromal cells and arguably demonstrated the most convincing restoration of the native stromal cell distribution. DAPI stained cross-sections showed excellent distribution of cells within the DC following 5 weeks culture. In addition, this report offers evidence that the stromal cells may be returning to their quiescent phenotype, using immunohistochemistry to stain for ALDH.

Attempts to improve the porosity of DCs were reported by Xiao *et al.* (2011) [74] in which a post-decellularization freeze drying process was used to disrupt the lamellae. In this investigation freeze dried constructs were seeded at a density of 2.5 × 10^5^ stromal cells/stroma using an intra-stromal injection, achieving a good distribution of stromal cells following 12 days of culture. Disappointingly, there was no non-freeze dried control in this experiment to reference, but histological evidence showed a clear difference to typical DC stroma.

#### 5.2.3. Endothelial Cells

Corneal endothelial cells have very limited proliferative capacity *in vivo*, dividing rarely in the adult cornea [223,224], thus wounding and trauma of the endothelium cannot be reversed. If there is a severe loss of corneal endothelial cells and the ability to pump water away from the cornea is lost, the only effective therapy is to replace the entire endothelium, usually with one from a cadaveric donor. A therapy which supports an intact endothelial layer, would be a major achievement and this could be performed using a DC. 

Human corneal endothelial cells can be isolated and cultivated *in vitro*, but long-term culture is difficult to achieve, with the exception of embryonic or very young donors [225,226,227,228]. *In vivo*, corneal endothelial cells are tightly adhered to the Descemet’s membrane and detaching cells from this substratum can be damaging due to prolonged enzyme exposure. Once detached, the cells need to be cultured on a refined substrate comprising key components of the basement membrane such as collagen type IV, laminin, fibronectin and chondroitin sulfate [229,230]. More recent developments have improved the prospects for endothelial cell culture with specialist medium that allows for improved cell survival [231,232,233,234]. This medium requires the presence of GFs such as fibroblast growth factor (FGF), epidermal growth factor (EGF) and nerve growth factor (NGF).

There is evidence suggesting the existence of corneal endothelial stem cells, present in the periphery of human cornea [235,236,237], that show promise as a cell source for corneal endothelial regeneration. Other sources of stem cells that show potential for corneal endothelial differentiation include MSCs [238], bone-marrow derived endothelial progenitors [239], neural crest cells [240] and corneal stromal stem cells [241]. If stem cells could be effectively differentiated into large numbers of corneal endothelial cells, these could subsequently be utilized to create a functioning endothelium on a substrate such as a DC.

Currently, relatively little work has been performed investigating the recellularization of the posterior surface of DCs with endothelial cells, predominantly due to difficulties associated with extracting and expanding primary endothelial cells [44,69,232]. Proulx *et al.* (2009) [232] have used freeze thaw cycles to devitalize human corneas, which were then seeded with primary expanded feline endothelial cells, of unknown passage, upon the Descemet’s membrane. After 24 hours of tissue culture, a complete endothelial monolayer had been achieved. The endothelial monolayer showed the expected morphology and expressed the function-related proteins ZO-1, Na^+^/K^+^-ATPase, and the Na^+^/HCO3^−^ co-transporter. 

### 5.3. *In Vivo* versus *ex Vivo* Recellularization

To date, groups have investigated recellularization of DCs through both *in vivo* implantation and subsequent recellularization; and *ex vivo* *via* primary cell extraction and expansion, before seeding onto the DC and implanting into the host. *In vivo* recellularization evidence is confined to epithelialization and stromal cell in-growth, as no published work has investigated the *in vivo* re-endothelialization of such scaffolds. In general, *in vivo* epithelialization occurs in most cases, rapidly and without complication. Epithelialization over grafts has also been reported in other constructs in both animal models and humans and is not foreseen to be a significant problem, that requires *ex vivo* handling [242]. The largest question appears to be whether or not stromal recellularization should be carried out *in vivo* or *ex vivo.* Implantation of a completely acellular construct naturally has advantages in its manufacturing simplicity and reduced immunogenicity, however *in vivo*,there have been few convincing examples to date of significant stromal cell repopulation. Those that have shown success have had to modify the matrix to increase porosity, disrupting the matrix, and even then significant infiltration is only seen over extended time periods [74]. *Ex vivo* seeding of stromal cells through intra-stromal injection has shown some encouraging results, and may offer a means to control the distribution and differentiation of the culture sensitive stromal cells. However, this approach is likely to be far more burdensome when it comes to clinical translation. The predominant issue with *ex vivo* seeding of cells is the possibility of inducing an immune response from the host due to the allogeneic cell source. However, the risk of this is equal to the risk of performing a normal allogeneic cadaveric donor graft.

## 6. Alternative Use of Human Decellularized Tissues for Toxicity Testing

Another potential avenue for the use of human DCs that have been cell seeded *ex vivo* is the production of a more relevant human cornea substitute for use in toxicity testing and the development of new ocular drugs, as a robust non-animal alternative. The complexity and uniqueness of the cornea often makes the development of ocular drugs particularly challenging. The cornea is sensitive to various irritants and many substances can cause serious irreversible damage to the cornea, including ocular drugs. Before potential new ophthalmic drugs can be used routinely, they have to undergo many rigorous toxicity and permeation tests. Pharmaceuticals, cosmetics, toiletries, household, industry, agricultural, and military products are all potential irritants to the eye [243]. In order to ensure that they are safe for their intended use, all manufactured consumer products and ingredients must be tested and eye irritation potential assessed. Eye toxicity tests are therefore required to provide information that ensures that products are safely manufactured and labeled. Currently, no reliable human corneal substitutes exist for drug and toxicity testing.

The current international standard assay for acute ocular toxicity is the rabbit *in vivo* Draize eye test [244]. The procedure involves the application of 0.1 mL (100 mg solid) test substance onto the eye of a conscious rabbit for 4 hours. The rabbits are observed for up to 14 days for signs of irritation including redness, swelling, discharge, cloudiness, blindness *etc*. [243]. The observed degree of irritancy allows chemicals to be classified, ranging from non/mildly irritant to strongly irritant. Draize testing is often criticized due to lack of repeatability and over-prediction of human responses [244], primarily due to interspecies differences. In addition, the test is often disapproved of by animal activist groups, due to animal stress. Moreover, research councils, government and independent organizations are working extensively on the replacement, refinement and reduction (the 3Rs) of animal use in research and testing [245]. Furthermore, EU directives and guidelines (Directive 2010/63/EU) [246] “*strongly encourage*” *in vitro* screening of all components prior to animal testing and encourage the use of alternative testing when possible or available.

As an alternative to *in vivo* testing, enucleated eye tests using isolated rabbit eyes were first introduced in 1981 by Burton *et al.* [247]. They are ethically advantageous with reduced costs. Corneal thickness, opacity and fluorescein retention [248] are tested to reveal adverse reactions to test substances. Eye irritation is primarily determined by the extent of initial injury, which correlates with the extent of cell death and ultimately the outcome of an irritant on an eye [244]. Generally, slight irritants damage the superficial epithelium, mild irritants penetrate further to damage the stroma and severe irritants penetrate through the cornea and damage the endothelium [244]. However, as with Draize testing, interspecies differences lead to discrepancies in irritation compared to human responses. 

Slaughterhouse waste has been investigated as an alternative tissue source [248]. Porcine corneas are often used for corneal testing [249], although chicken enucleated eye tests (CEET) are widely accepted to be the most reliable and accurate slaughterhouse tissue for assessing the eye irritation potential of test materials [248]. CEET are often used as a pre-screen for Draize testing. Although, despite promising outcomes, the *in vivo* Draize testing results still overrule *ex vivo* results if discrepancies occur. 

*In vitro* toxicity testing using cultured cells, often on collagen hydrogels, are advantageous compared to *in vivo* and *ex vivo* testing because they are relatively inexpensive, are simple and can be rapidly manufactured. Rabbit corneal epithelial (RCE) cells have been cultured on collagen hydrogels using air-liquid interface techniques. The RCE model [250] aims to mimic the native rabbit corneal epithelium. To validate the model, thirty chemicals with known degrees of eye irritation (from Draize testing) have been tested. Eye irritation potency can be estimated by using colorimetric MTT assays as a measurement of viability. However, the RCE model is somewhat limited because it only models the epithelial layer and cannot be used to determine the possible effects of drugs penetrating the stroma and endothelium.

Although promising results have been obtained from both enucleated eye testing and RCE models, they share the common disadvantage of interspecies differences regarding anatomy and physiology. Such differences produce discrepancies in permeation studies and toxicity tests [251,252]. Pathogens are often species specific and this regularly causes new drugs to fail in clinical trials [253]. In response, human cell based culture models are becoming more established, but the principal hurdle is the successful manufacture of an *in vitro* cornea with comparable barrier functions to the native cornea. Thus, there is still a growing need for credible, human-derived *in vitro* models [253].

Griffith *et al.* (1999) [36] produced the first functional equivalent of a human cornea using immortalized human corneal cells. A collagen-chondrotin sulfate substrate cross-linked with glutaraldehyde was used as a tissue matrix. Initially, a thin layer of endothelial cells were grown in a culture dish. Keratocytes and support proteins were added, before finally adding the final epithelial layer. Gross morphology, transparency and histology were reported to be similar to that of a natural cornea. Tests performed using mild detergents determined that the constructed cornea had a similar gene expression and wound-healing response when compared to human eye-bank corneas. 

In the wake of this work, Reichl *et al.* (2001) [254] claimed to have successfully manufactured a human corneal equivalent for *in vitro* drug permeation studies by culturing all three corneal cell types in a collagen hydrogel matrix. Three reagents commonly used in ophthalmic drugs to treat glaucoma and inflammatory diseases were tested and permeation data obtained was compared with those from excised porcine cornea and a porcine cornea construct [251,252]. The human cornea construct had similar epithelial barrier properties to a native cornea with only small ultrastructural differences, possibly due to lack of tears and blinking. For all reagents examined, there was increased permeability in the TE constructs compared to the exercised porcine cornea, although the differences were relatively small. Unfortunately, there was no data available for comparison with an excised human cornea, as in the studies by Griffith *et al.* (1999) [36]. 

The MatTek Corporation has developed a commercially available 3D corneal epithelial model based upon human derived epidermal keratinocytes from human foreskin [28,255], marketed as EpiOcular^TM^. Although it is been used by numerous cosmetic companies in place of Draize testing, as of yet EpiOcular^TM^ has not been formally validated [255] as it is unable to predict responses of chemicals that affect the lower layers of the cornea, or that are dependent upon epithelial-stromal cellular interactions [28]. 

Despite numerous screening tests, currently there is no validated *in vitro* ocular irritation test to replace the heavily criticized use of animals. This is partly due to a lack of understanding of the underlying mechanisms of eye irritation [250], a possible the lack of innervation [23] and an apparent reluctance of regulatory bodies to accept new *in vitro* corneal constructs. However, recent European directives have prohibited the use of laboratory animals in toxicity testing, particularly the use of the Draize eye irritancy test [37], thus the need for alternative testing is crucial, particularly in the development of new ophthalmic drugs. Human DCs that have been regenerated using specific, defined cell lines have the potential to be the primary method of toxicity testing for many of the requirements discussed above. They would offer a full-thickness, functional tissue that is ethically advantageous and species specific, capable of accurately predicting human responses of a whole spectrum of irritants, ranging from mildly to severely irritant, in a reliable, reproducible manner.

## 7. Conclusions

The limited availability of suitable corneal donor tissue has led to the development of alternative corneal equivalents including KPros and TE corneas. It has been impossible thus far to replicate the structural complexity of the native cornea *in vitro* and artificial equivalents only simulate physical characteristics, topography and lack intrinsic functionality [3]. Human DCs theoretically provide an authentic scaffold for corneal tissue reconstruction that can maintain structural complexity and provide authentic cell-cell and cell-matrix interactions, which are pivotal for cell differentiation and conservation of specialized functions.

There is currently no standard technique for decellularization of the cornea and a range of protocols have been attempted with varying levels of success. The important factor in any decellularization protocol is achievement of a balance of maximum cell debris removal with minimal structural disruption. This requires more investigation with the ultimate aim of producing a standardized and repeatable decellularization protocol. There are many options for both destructive and non-destructive characterization of a DC, with non-destructive techniques showing potential as part of manufacturing processes. The possibility of incorporating cells within the human DCs has been considered, but there is as of yet no evidence to show the necessity of this process.

Ultimately, although many challenges are ahead, human DCs may provide a promising alternative to donor corneas in addition to becoming an alternative method of toxicity testing and allowing the study of corneal biology and potential new drug development.

## 8. Perspectives

Although bioscaffold design has significantly advanced in recent years, we are yet to achieve a suitable corneal equivalent that is capable of mimicking the complex lamellar structure of the native tissue. Simply put, the most suitable matrix for tissue engineered corneas is the corneal tissue itself. 

The most attractive strategy at current for development of a clinical product would be the production of a DC seeded with stromal cells. Through intra-stromal injection, one could ensure suitable distribution of stromal cells within the matrix, and in an ideal situation, control stromal cell phenotype through conditioning with specific medium. 

At the time of this review there were three registered clinical trials involving DCs. Two of the three reported investigation of decellularized grafts from human source, and the other from porcine origin [256]. The clinical data from the solitary completed study has now been published, in a peer reviewed journal, with promising results [170]. This further validates the vast potential of DCs, demonstrating the vital need for further research and exploitation. 
